# Effect of Low-Dose Line-Spectrum and Full-Spectrum UV on Major Humoral Components of Human Blood

**DOI:** 10.3390/molecules28124646

**Published:** 2023-06-08

**Authors:** Madina M. Sozarukova, Nadezhda A. Skachko, Polina A. Chilikina, Dmitriy O. Novikov, Elena V. Proskurnina

**Affiliations:** 1 Kurnakov Institute of General and Inorganic Chemistry, Russian Academy of Sciences, Leninsky av., 31, 119991 Moscow, Russia; 2 Department of Plasma Power Plants, Bauman Moscow State Technical University, 2-nd Baumanskaya, 5, 105005 Moscow, Russia; nadyans96@mail.ru (N.A.S.); p.chilikina@gmail.com (P.A.C.); zerooo@list.ru (D.O.N.); 3 Research Centre for Medical Genetics, ul. Moskvorechye 1, 115522 Moscow, Russia; proskurnina@gmail.com

**Keywords:** ultraviolet, mercury lamp, flash xenon lamp, blood plasma, human serum albumin, uric acid, antioxidants, chemiluminescence

## Abstract

Ultraviolet blood irradiation (UVBI) is an alternative approach to the treatment of infectious diseases of various pathogeneses. Recently, UVBI has attracted particular interest as a new immunomodulatory method. Experimental studies available in the literature demonstrate the absence of precise mechanisms of the effect of ultraviolet radiation (UV) on blood. Here, we investigated the effect of UV radiation of line-spectrum mercury lamp (doses up to 500 mJ/cm^2^) traditionally used in UVBI on the major humoral blood components: albumin, globulins and uric acid. Preliminary data on the effect of various doses of UV radiation of full-spectrum flash xenon lamp (doses up to 136 mJ/cm^2^), a new promising source for UVBI, on the major blood plasma protein, albumin, are presented. The research methodology included spectrofluorimetric analysis of the oxidative modification of proteins and analysis of the antioxidant activity of humoral blood components by chemiluminometry. The effect of UV radiation on albumin caused its oxidative modification and, accordingly, an impairment of the transport properties of the protein. At the same time, UV-modified albumin and γ-globulins acquired pronounced antioxidant properties compared to native samples. Uric acid mixed with albumin did not protect the protein against UV-induced oxidation. The flash full-spectrum UV qualitatively had the same effect on albumin as line-spectrum UV did, but an order of magnitude lower doses were required to achieve comparable effects. The suggested protocol can be used for selecting a safe individual dose for UV therapy.

## 1. Introduction

Ultraviolet blood irradiation (UVBI) was widely used in the 1940s and 1950s as a therapy for various diseases, such as viral infections, bacterial infections, inflammation, even for treatment of liver and biliary disease [1,2]. The current increased interest in the therapeutic potential of UVBI is due to new viral infections, including Ebola, SARS, MERS and SARS-CoV-2, and their resistance to existing antiviral drugs, vaccines and antibacterial medications [2]. The anti-inflammatory effect of UV radiation can be used in immunomodulatory therapy [3,4]. As ultraviolet radiation is not safe, its medical applications must be based on a clear balance of benefit and risk. An assessment of risk and benefit can only be made on the basis of the mechanisms of action. Therefore, despite the advantages of UVBI, a lack of understanding of the mechanisms of action and the development of efficient antibiotics have reduced the practical application of ultraviolet blood irradiation [1].

Both cellular components and non-cellular blood components—cells and plasma with its water- and fat-soluble low-molecular-weight antioxidants, proteins and lipids—are affected by UV. Thus, UV irradiation (UVI) modulates the functions of erythrocytes, leukocytes and platelets, which has been demonstrated by numerous in vitro experiments. The review describes the main effects observed of exposure to UVBI on blood cells [1].

UV radiation induces oxidative stress [5]. When blood is irradiated, plasma antioxidants are the first protective targets of UV light, whereas blood cells are the effector targets. Blood plasma contains active water- and fat-soluble antioxidants, and the compensation of acute oxidative stress (OS) is one of its most important functions. A major water-soluble low-molecular-weight plasma antioxidant is uric acid [6]. Albumin resulting from one thiol group (mercaptoalbumin) shows strong antioxidant properties [7,8], while little is known about the antioxidant properties of globulins.

Active research works on the effect of UV radiation on albumin were conducted in the middle of the last century [9,10,11]. The binding of UV-irradiated albumin with DNA [12], bilirubin [13] and changes in its conformation [10] were studied. Recent works have focused on the study of new objects, such as protein films [14,15,16], because of their high biocompatibility and biodegradability. Moreover, they have similarities with the extracellular matrix. Researchers discovered the unique adhesive properties of UV-irradiated albumin films [14]. As a result of UV irradiation, a destruction of the α-helix structure, breaking of peptide bonds and increase in hydrophilicity were observed in protein molecules. Human serum albumin (HSA) was selected for constructing protein films, which keep their functional properties [15]. Albumin is capable of preventing the adsorption of other proteins and cell adhesion and binds a large number of various ligands [15,16].

Most studies have been performed with relatively high doses of UV, leading to a marked change in the structure of the protein, up to denaturation, but significantly lower doses are used for medical purposes. There are some works that show, for example, that the binding activity of albumin increases with the UVI of autoblood at therapeutic doses [17]. However, the available data are represented in early studies and are very scarce, which makes it relevant to study the mechanisms of low-dose effects of UV on the major water-soluble plasma antioxidants: albumin and uric acid.

UV phototherapy uses both medium-wavelength rays (UV-B, 280–320 nm) and long-wavelength rays (UV-A II, 320–340 nm and UV-A I, 340–400 nm). The wavelength determines the penetration ability and biological effect. The long-wavelength spectrum induces early apoptosis, while the medium-wavelength spectrum induces late apoptosis [18]. In most cases, a mercury lamp generating a line spectrum is used. The main line of a mercury lamp of 254 nm falls into the absorption maximum of intracellular chromophores (DNA). As a result, photoproducts are formed (pyrimidine dimers), which affect the cell cycle. The doses used are approximately 100 mJ/cm^2^ for UV skin therapy (initial dose, with subsequent increases of 100 mJ/cm^2^) [19] and approximately 10 mJ/cm^2^ when irradiating blood [20]. The study of UVBI continues to this day. Relatively recently, promising therapeutic effects of UVBI in a combination therapy with antibiotics have been published [21,22,23]. In particular, UVBI has been successfully promoted in recent years in phase II clinical trials for the treatment of hepatitis C infection [22].

The traditional sources of radiation for UVBI are low-pressure mercury lamps operating in continuous mode. The line emission spectrum of mercury lamps eliminates the possibility of exposure to all components of the blood. Among other significant disadvantages of mercury lamps, a relatively low radiation power and insecurity render them unsafe. Recently, promising sources for UV therapy have been developed. These are high-intensity flash xenon lamps with a wide spectral range from UV-C to near IR. Bactericidal efficiency in the 205–315 nm range is 10–13% of the total xenon lamp radiation. The main advantages of flash sources are the continuous emission spectrum and high peak power (5–50 MW) at a pulse duration of 1–20 µs [24]. The short time exposure is also a distinctive feature of the xenon source. It coincides in order of magnitude with the lifetime of many excited states and characteristic times of chemical reactions, but it is not comparable with the corresponding times of biological reactions in the body. Due to the high intensity of flash UV sources, photo-oxidation reactions proceed mainly through a chain, highly branched mechanism; therefore, the quantum yields of photoprocesses are significantly increased. As a consequence, the specific energy consumption is reduced, and the productivity of the process is increased.

Here, we aimed to investigate in detail the oxidative effects of low-dose line-spectrum UV from a mercury lamp. In addition, this current work presents the preliminary results of the oxidative effects of a high-intensity broadband UV source: a pulsed xenon lamp. The results obtained confirm the prospects for further study of the effects of a pulsed xenon lamp. The major water-soluble humoral UV chromophores of the blood (albumin, globulins and uric acid) were chosen as targets.

## 2. Results and Discussion

Our study includes the following steps:

1. study of the effects of low-dose line-spectrum UV (mercury lamp) on the fluorescence of aromatic amino acids and albumin, oxidative modification of albumin, transport properties of albumin, antioxidant properties of albumin, globulins, uric acid, a mixture of uric acid and albumin, and blood plasma;
2. study of the effects of low-dose full-spectrum UV (flash xenon lamp) on the oxidative modification of albumin, its transport and antioxidant properties.

### 2.1. Mercury Lamp as a Source of UV Radiation

#### 2.1.1. Contribution of Tyrosine and Tryptophan to UV-Induced Oxidative Modification of HSA

Serum albumin is one of the main targets of UV irradiation in blood plasma due to eighteen tyrosine residues and one tryptophan residue. Previously, we showed that UV irradiation of these amino acids leads to photochemical reactions resulting in the formation of dityrosine and dioxyphenylalanine [25]. Dioxyphenylalanine exhibited pronounced antioxidant potential, which probably compensated for UV-induced oxidative stress [25].

**Figure 1:**
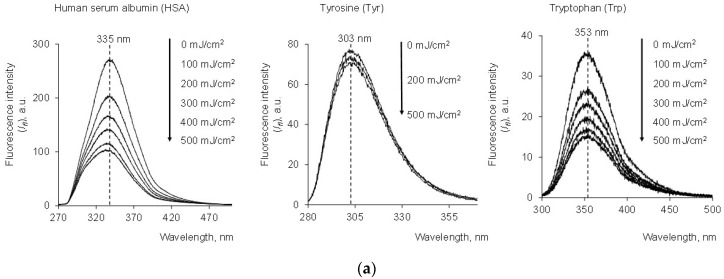

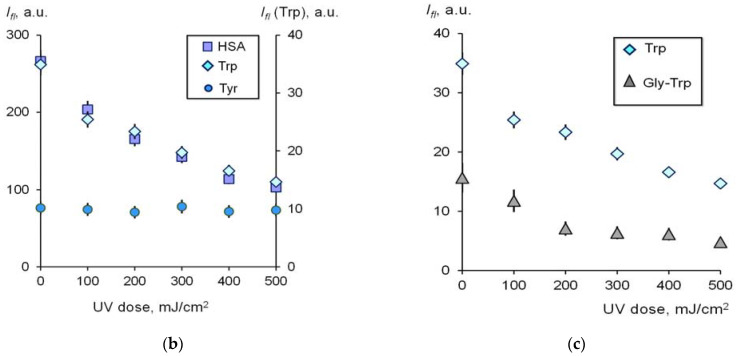
(**a**) Fluorescence spectra of HSA (*c* = 1.5 μM) after exposure to UV (254 nm; the doses are shown in the figure); (**b**) change in fluorescence intensity (*I_fl_*) vs. UV dose for HSA (1.5 μM), Tyr (27 μM) and Trp (1.5 μM) at wavelengths of 335 nm, 303 nm and 353 nm, respectively; (**c**) change in *I_fl_* vs. UV dose for Trp (1.5 μM) and Gly-Trp (1.5 μM) at 353 nm and 355 nm, respectively; λ_ex_ = 260 nm.

We examined the irradiation of serum albumin, tyrosine, tryptophan and glycyltryptophan through various low doses of mercury lamp UV with registration of fluorescence spectra (Figure 1a,b):

For both HSA and Trp solutions, the fluorescence intensity (*I_fl_*) decreased with increasing energy exposure (mJ/cm^2^). The analytical signal of Tyr practically did not change because it was characterized by an order of magnitude less effective absorption. Therefore, oxidative structural modification of HSA can be determined by photolysis of tryptophan. In this case, this was also supported by changes in the fluorescence spectra recorded for the dipeptide with a glycyl residue (Gly-Trp) (Figure 1c). However, it should be noted that this decrease in tryptophan fluorescence does not always indicate structural changes in the protein. Changes in tryptophan fluorescence intensity can be caused by local chemical modification of this amino acid. At the same time, these changes may not affect the structure of the protein.

At equal concentrations of tryptophan and albumin, the fluorescence intensity for albumin was several times greater, which is associated with the quenching of tryptophan fluorescence in aqueous solution. However, the doses at which fluorescence halving occurred for tryptophan (368 mJ/cm^2^) and albumin (353 mJ/cm^2^) were approximately equal.

Photofluorescence spectra reflect the conformational changes in the albumin macromolecule caused by UV light, as demonstrated elsewhere [26,27,28,29]. The authors proved the maximum decrease in fluorescence intensity occurred at 1510 J/m^2^, which was indicative of rearrangements affecting the Trp residue and its microenvironment [29]. Another study was devoted to the study of the optical properties of bovine serum albumin after exposure to UV in the presence of β-carotene [30]. The authors suggested that phototransformation of the protein molecule, leading to a change in its conformation at low doses of UV, contributed most to the formation of a complex between albumin and β-carotene.

The effect of UV (254 nm) on HSA in the presence of water-soluble antioxidants (gallic acid, nicotinic acid and ascorbic acid) was analyzed through changes in absorption spectra [31]. As reported in Ref [28], the ratio between absorbances at 255 nm and 280 nm (A_max_/A_min_) was useful for estimating the UV-induced structural changes in the albumin molecule. A decrease in A_max_/A_min_ can be assigned to chemical changes in the aromatic amino acid residues (Trp, Tyr and phenylalanine (Phe)) or reorganization of the regions where they are located [10]. Trp, Tyr and Phe, along with cysteine (Cys) residues, are the main targets of UV radiation. Their absorption of UV light results in many chemical and/or physical events, causing a partial or complete loss of protein activity. Moreover, Tyr and Phe in the excited state are capable of transferring energy to tryptophan, enhancing the undesirable effects of UV irradiation. As noted by the authors [31], the secondary effects of UV-B were associated with the formation of reactive oxygen species (ROS) and oxidative damage with changes in its secondary and tertiary structures [32]. As was demonstrated in Ref [31], the A_max_/A_min_ ratio remained constant, which proved the absence of photochemical changes in the aromatic amino acid residues, regardless of the presence of antioxidants in the environment. However, exposure to UV radiation led to a gradual increase in absorbance at 300–340 nm, which may be associated with the formation and growth of potentially immunogenic protein aggregates, and all three antioxidants studied prevented the aggregation process. Since aggregation is caused by ROS formed in aqueous solution under UVI, the authors concluded that the observed differences in HSA protection are due to the radical intercepting properties of these compounds (ascorbic acid turned out to be the most highly effective compound). However, interactions of antioxidants with the protein molecule may also be important, since there is increasing evidence of a lability of the three-dimensional structure of albumin, which permits cooperativity and allosteric modulation in interactions with various substances, usually characteristic of multimeric proteins [33].

To summarize, UV radiation primarily causes tryptophan phototransformations, which leads to changes in the high-level spatial organization of HSA. These changes can affect the ligand-binding properties of albumin, which is the main transport protein in blood. Next, we examine the effect of UV on the transport properties of HSA.

#### 2.1.2. Effect of UV on the Transport Properties of Human Serum Albumin

Serum albumin provides transport for various substances (fatty acids, metal ions, bilirubin, hormones and drugs) due to reversible binding of ligands in the so-called “hydrophobic pockets” [34]. These are areas containing many non-polar amino acids, which form the binding sites for molecules. These sites also provide the enzymatic activity for albumin: esterase (Sudlow site I) and pseudoesterase (irreversible substrate–enzyme binding, Sudlow site II) [33].

**Figure 2:**
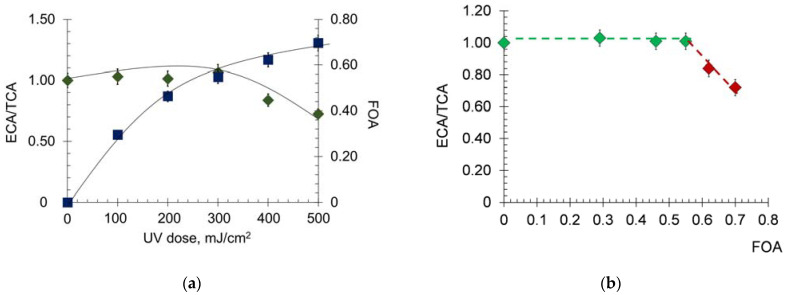
(**a**) The changes in FOA and the ECA/TCA vs. UV dose for HSA (6.6 μM); (**b**) correlation of the ECA/TCA and FOA parameters.

The fluorescent probe K-35 (N-(carboxyphenyl)imide 4-(dimethylamino)naphthalic acid) allows a selective study of changes in the drug site II near which the tryptophan residue is localized. The effective concentration of albumin (ECA) is calculated as the ratio of *I_fl_* HSA before irradiation to *I_fl_* HSA after irradiation in the presence of a K-35 probe at 518 nm. TCA is a total concentration of albumin, and FOA is the fraction of oxidized albumin. We calculated the ECA/TCA ratio as a parameter of albumin transport capacity and FOA as a parameter of oxidative modification (Figure 2a,b):

With increasing UV radiation dose, ECA/TCA gradually decreased, and the fraction of oxidized protein increased. When FOA exceeds 0.5, albumin transport properties begin to fall sharply (Figure 2b).

A review by Oettl et al. focuses on the effects of oxidative modification on the binding properties of albumin, ranging from no effect to a decrease or increase in albumin affinity, depending on ligands and external factors (the method of oxidative modification) [35]. Most of the available studies of the ligand-binding characteristics of HSA were performed with oxidized albumin in vitro, using different oxidation methods and assays. Albumin was incubated with ascorbic acid in the presence of oxygen and trace metals, hydrogen peroxide, chloramine T, glucose, cystine or homocysteine and NO. The content of carbonyl, -SH and S-nitro groups, mercapto- and non-mercaptoalbumin fractions, formation of tyrosine dimers and glycosylation products were evaluated as indicators of oxidative modification of HSA. Free ligands were quantified using a variety of methods, from high-performance liquid chromatography to titration. The binding capacity of HSA (binding constants) was evaluated using spectrofluorimetry, circular dichroism, nuclear magnetic resonance or dialysis. The change in the ligand-binding ability of HSA depended on the oxidant nature. The binding of warfarin, which is the ligand for site I, was not affected by the ascorbic acid/FeCl_2_-induced HSA modification. In contrast, albumin oxidatively modified by chloramine T or the protein of patients on hemodialysis showed decreased affinity for warfarin compared with native HSA or plasma albumin of a control cohort. A slight increase in warfarin binding of glycosylated recombinant human albumin was unexpected. On the other hand, the binding of ketoprofen (a non-steroidal anti-inflammatory drug), the ligand for drug site II, was decreased in all methods of oxidative modification of HSA, and this was also observed for the albumin of patients on hemodialysis. It should be noted that, as a rule, drug site II is more sensitive to oxidation [35]. Thus, oxidative modifications of albumin affect the transport function of the molecule in different ways. Moreover, the pharmacokinetics also changes, since oxidized species are rapidly eliminated from the bloodstream.

Thus, we can conclude that the nature and extent of changes in the transport properties of albumin are essentially determined by the nature of the oxidant. The changes can affect both or one of the two drug sites of the protein. From these experiments, it should be concluded that low-dose UV radiation causes a decrease in the transport properties of albumin as a result of oxidative modification when the fraction of oxidized albumin becomes 50% or more.

#### 2.1.3. Antioxidant Capacity of Albumin

The scavenger of radicals in serum albumin is the SH group Cys-34, which provides a major pool of active thiols in blood plasma [36]. Two sulfur-containing residues in HSA, Met and Cys, account for 40–80% of the total antioxidant activity of albumin, which is responsible for more than 70% of the free-radical-scavenging activity in serum [7]. Albumin, along with uric acid, provides the main water-soluble antioxidant potential for blood plasma [37].

**Figure 3:**
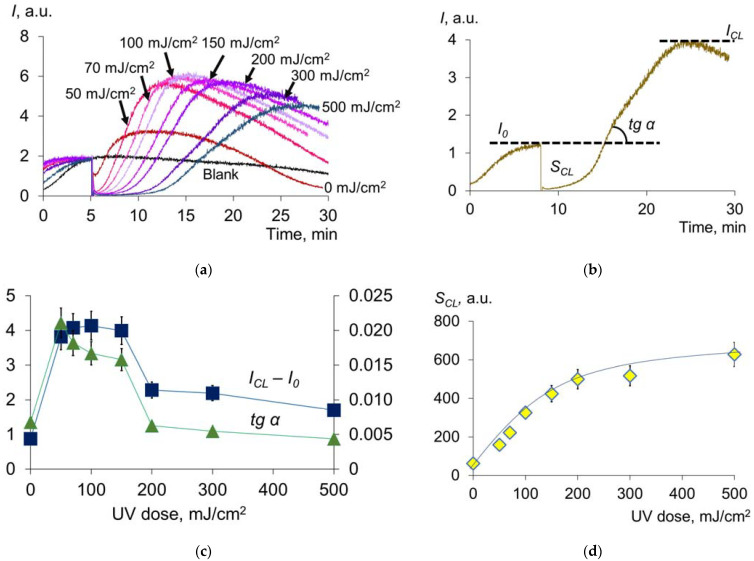
(**a**) Chemiluminograms of HSA (0.66 μM) after UV irradiation (254 nm; doses are shown in the figure) under the system of PBS (100 mM, pH 7.4) + ABAP (2.5 μM) + luminol (2.0 μM); (**b**) investigated parameters of the chemiluminogram: *I*_0_—initial luminescence level, *I_CL_*—a new steady-state level after the addition of albumin, *tg α*—the tangent of the chemiluminescence rise angle; (**c**,**d**) functions of *I_CL_* − *I*_0_, *tg α* and *S_CL_* (area of chemiluminescence suppression) vs. UV doses (254 nm).

We evaluated the antioxidant potential of HSA with luminol-enhanced chemiluminometry using thermal-induced decomposition of the diazo compound as a source of free radicals (Figure 3a).

The chemiluminograms were characterized by complex kinetics, each component of which can be described by quantitative indices (Figure 3b) [38], where *I_CL_*—a new level of steady-state luminescence after the addition of albumin, *tg α*—the tangent of the chemiluminescence rise angle and *S_CL_*—area of chemiluminescence suppression. According to our non-published data, the difference *I_CL_* − *I*_0_ reflects the level of the non-oxidized group -SH, i.e., the concentration of mercaptoalbumin. The area of chemiluminescence suppression characterizes the number of intercepted radicals and reflects the antioxidant capacity. The precise physical meaning of the luminescence tangent has yet to be elucidated, but this parameter also varies with dose. Preliminary studies were carried out in this area. The chemiluminescence curves of blood plasma and a mixture of basic water-soluble antioxidants modeling blood plasma antioxidant pool were compared. According to the results obtained, we can assume that the tangent of the chemiluminescence rise angle (*tg α*) characterizes the oxidation of albumin: the more oxidized the albumin, the lower the value of tg α.

A sharp increase and subsequent decrease in *I_CL_* at doses up to 200 mJ/cm^2^ (Figure 3c) were observed, after which the indices remained practically unchanged. Apparently, at a dose of 200 mJ/cm^2^, complete oxidation of the thiol group -SH occurs. The dose dependence of *tgα* behaves similarly. The antioxidant capacity *S_CL_* (the effect of strong antioxidants) increases with increasing dose (Figure 3d). Consequently, increased oxidative modification of HSA leads to an increase in the total antioxidant potential [39]. This increase is due, among other things, to the formation of *N*-formylkinurenine and dihydroxyphenylalanine, which have antioxidant activity [25].

**Figure 4:**
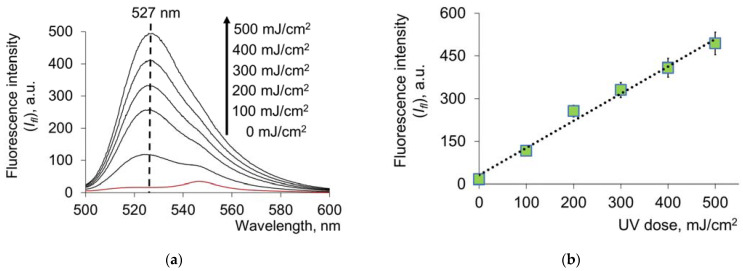
(**a**) Fluorescence spectra of HSA (*c* = 1.5 μM) with the reagent for the determination of free –SH groups after exposure to UV radiation (254 nm; doses are shown in the figure); (**b**) change in fluorescence intensity (*I_fl_*) from UV doses at a wavelength of 527 nm. Registration of fluorescence at λ_ex_ = 490 nm.

A free SH group is the most sensitive to free-radical processes. HSA contains 34 cysteine residues, forming intramolecular disulfide bonds (bridges) and one free thiol group in the Cys34 position. Under oxidation, the SH group forms disulfide bonds with free cysteine and other thiols in the plasma or oxidizes to sulfenic and sulfinic acids [40,41]. Thus, 17 disulfide bonds and the free SH group present in the structure of HSA contribute to the strengthening of antioxidant properties when exposed to UV. This was proved by the fluorescence spectra of UV-modified HSA using a commercial thiol assay kit. As the UV dose increased, an increase in fluorescence at 527 nm was observed (Figure 4a,b):

Exposure to UV leads to a dose-dependent increase in the content of free thiol groups in the protein as a result of disulfide bridging, which in turn is reflected in the antioxidant profile of HSA; namely, it leads to an increase in its radical scavenging properties. Using a standard solution of glutathione, a calibration dependence was obtained, and the content of the SH groups was determined (Table 1):

Due to the presence of a free SH group, albumin forms the largest thiol pool in the blood [35]. Depending on the redox state of this group, HSA exists in two forms: reduced albumin (mercaptoalbumin) and oxidized albumin (non-mercaptoalbumin) [42,43]. Several sulfhydryl/disulfide pairs exist in plasma, including Cys34 albumin [35]. In healthy humans, about 70–80% of the Cys34 in albumin contains a free sulfhydryl group (human mercaptoalbumin, HMA); 25% of Cys34 forms disulfides with small sulfhydryl compounds, such as other cysteine, homocysteine or glutathione (human non-mercaptoalbumin-1, HNA1); and a small fraction of Cys34 is more strongly oxidized to sulfinic or sulfonic acid (human non-mercaptoalbumin-2, HNA2) [44]. The ratio of these types of protein may act not only as a useful biomarker of the body’s redox status, but it is also thought to influence the pathogenesis and progression of several diseases, among them, liver damage, renal failure, diabetes, atherosclerosis and other cardiovascular diseases [43].

To summarize this section, low-dose irradiation leads to an increase in the antioxidant capacity of albumin, most likely due to the breaking of disulfide bridges. Using the values of the *S_CL_* parameter, the antioxidant capacity of a 1 µmol/L albumin solution after exposure to a dose of 100 mJ/cm^2^ was estimated in terms of the concentration of the water-soluble vitamin E analog Trolox, µmol/L (see Supplementary Materials). According to the estimates, the ability of albumin irradiated with a dose of 100 mJ/cm^2^ to scavenge free radicals is, on average, three times lower than that of Trolox.

#### 2.1.4. Changes in Fluorescence and Antioxidant Activity of γ-Globulins under UV Radiation

**Figure 5:**
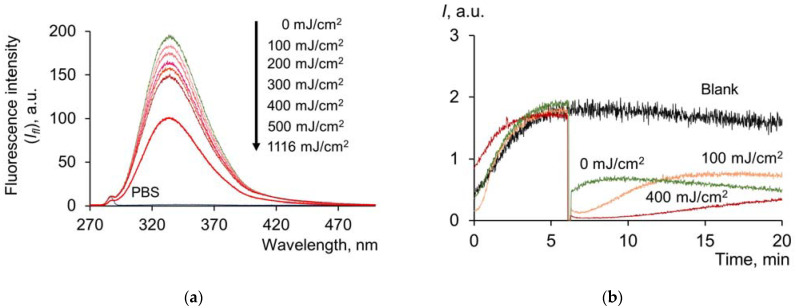
(**a**) Fluorescence spectra (λ_ex_ = 260 nm) of γ-globulins solutions (*c* = 0.66 μM) after exposure to UV (254 nm); (**b**) chemiluminograms of γ-globulins solutions (*c* = 0.66 μM) under the system of PBS (100 mM, pH 7.4) + ABAP (2.5 μM) + luminol (2.0 μM).

One of the important components of blood, along with HSA, are globulins. The amount of globulins in the plasma is comparable to the levels of HSA. A dose-dependent decrease in the intensity of tryptophan fluorescence was obtained for globulins by spectrofluorimetry (Figure 5a):

It should be noted that for γ-globulin and albumin solutions with comparable concentrations, it was determined that the tryptophan fluorescence of albumin is more sensitive to UV irradiation. The half quenching of fluorescence occurs at a dose of 450 mJ/cm^2^ for albumin, while for γ-globulins, this value is 1116 mJ/cm^2^. Presumably, such difference may be due to the nature of proteins, namely, the different degree of screening of tryptophan residues in the protein structure. Approximately 95% of the tryptophan residues of globulins are located in the inner regions of the globule.

The effect of UV radiation on the antioxidant potential of γ-globulins was also analyzed. The chemiluminescence curves (Figure 5b) are fundamentally different from the three-phase albumin chemiluminescence curves. The addition of globulins to the system with alkylperoxyl radicals led to a decrease in the signal at a new stationary level. This activity is typical for weak antioxidants. The kinetics of the antioxidant action of globulins after UV irradiation is characterized by a significant decrease in chemiluminescence, almost to zero values. Afterward, a slow rise in the chemiluminescence curve to a new stationary level was observed. All of this points to the formation of products with a more pronounced antioxidant activity after UV irradiation of globulins.

Thus, the antioxidant capacity of globulins increases as a result of UV irradiation.

#### 2.1.5. Effect of UV on the Antioxidant Properties of Uric Acid

**Figure 6:**
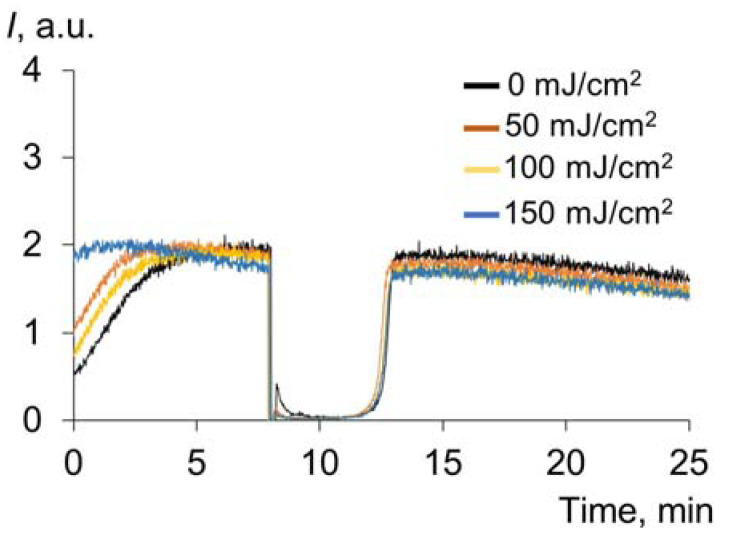
Chemiluminograms of uric acid (0.33 μM) after exposure to UV (254 nm) (doses are shown in the figure) under the analytical system of PBS (100 mM, pH 7.4) + ABAP (2.5 μM) + luminol (2.0 μM).

Another important water-soluble plasma antioxidant is uric acid (UA), which is characterized by UV absorption [45]. The chemiluminograms for UA after exposure to UV are shown in Figure 6. There was no effect of UV irradiation on the antioxidant capacity.

Uric acid is an antioxidant, which is capable of scavenging superoxide radical anion, hydroxyl radical and singlet oxygen. It chelates the transition metals and blocks the nitrosylation reaction of tyrosine residues through peroxynitrite (nitrotyrosine formation) of the proteins [46]. It is reported that uric acid can prevent the degradation of extracellular superoxide dismutase (SOD3), an enzyme critical for maintaining endothelial and vascular function [47]. The urate radical formed is much less reactive than the usual pro-oxidants and can be rapidly reduced by the ascorbate [48]. Elevated serum uric acid levels in patients with cardiovascular disease may reflect a compensatory mechanism to counteract oxidative stress, but the correlation of high uric acid with poor clinical outcomes is still unclear [49,50].

The studies indicate the dual activity of uric acid, which can act both as an antioxidant and a pro-oxidant, stimulating the proliferation of vascular smooth muscle cells and inducing endothelial dysfunction [6,50,51,52,53,54]. Moreover, uric acid can play the role of a signaling molecule for the immune system. The authors showed that uric acid and the cytosolic protein NLRP3 are produced in the skin of mice in response to UV [55]. In vitro and in vivo experiments confirmed that immunosuppressive doses of UV induce uric acid formation, suggesting that a possible trigger for this process may be the ability of UV-B to increase xanthine oxidase activity in keratinocytes. Thus, the local concentration of uric acid in the tissue plays a key role in determining whether it contributes to enhancing uric acid (at high doses) or inhibiting (at low doses) adaptive immune responses.

As a result, uric acid does not change the antioxidant potential, but it might have a protective effect on albumin. The following experiments were conducted to study the antioxidant potential of albumin under UV irradiation in the presence of uric acid.

#### 2.1.6. Effect of UV on Antioxidant Properties of Uric Acid and Albumin Mixture

**Figure 7:**
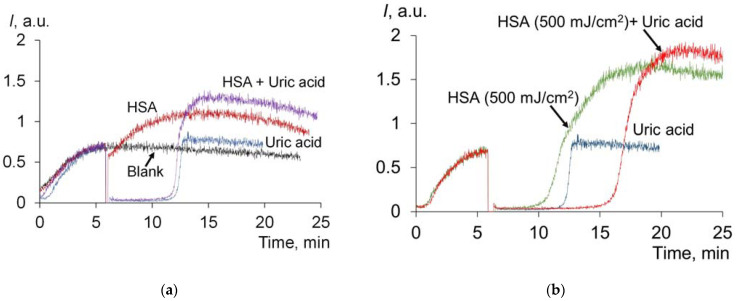
(**a**) Chemiluminograms of HSA (6.6 μM), uric acid (4.9 μM) and a mixture of HSA (6.6 μM) + uric acid (4.9 μM); (**b**) chemiluminograms of HSA (6.6 μM), uric acid (4.9 μM) and a mixture of HSA (6.6 μM) + uric acid (4.9 μM) after exposure to HSA with UV (254 nm; doses are shown in the figure). The analytical system contains PBS (100 mM, pH 7.4) + ABAP (2.5 μM) + luminol (2.0 μM).

**Figure 8:**
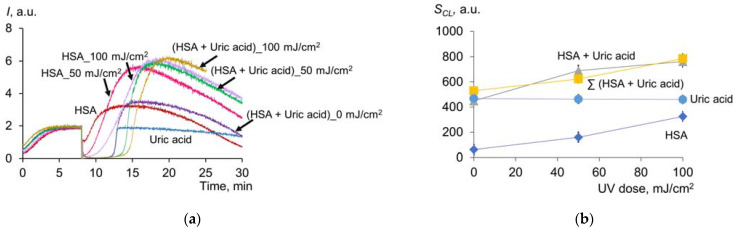
(**a**) Chemiluminograms of HSA (6.6 μM), uric acid (4.9 μM) and a mixture of HSA (6.6 μM) + uric acid (4.9 μM) after exposure to UV (254 nm; doses are shown in the figure). Curves were obtained under the system of PBS (100 mM, pH 7.4) + ABAP (2.5 μM) + luminol (2.0 μM); (**b**) dependences of *S_CL_* on UV dose (254 nm).

We mixed HSA with uric acid in concentrations corresponding to the actual amounts of these compounds in the blood plasma in healthy patients. In one series, pre-irradiated albumin was added to uric acid (Figure 7). In the second series, a mixture of uric acid and HSA was exposed to UV (Figure 8a):

Without irradiation, the chemiluminogram of the mixture of HSA + uric acid is essentially the sum of the effects of HSA and uric acid (Figure 7a). Irradiated HSA is characterized by strong antioxidant properties. In this case, the antioxidant potential of the mixture is also equal to the sum of antioxidant potentials of irradiated HSA and uric acid (Figure 7b): *S_CL_* (HSA, 500 J/cm^2^) = 140 ± 10 arb.u., *S_CL_* (UA) = 271 ± 12 arb.u., *S_CL_* (HSA (500 mJ/cm^2^) + UA) = 421 ± 25 arb.u.

The *S_CL_* data (Figure 8b) for the irradiated mixture of HSA + UA and a sum of the chemiluminescence suppression areas for the UV-modified individual solutions of HSA and UA are approximately comparable within the experimental error. Thus, although uric acid absorbs UV radiation, in aqueous solutions, it does not affect the UV-induced change in the antioxidant properties of albumin.

The question arises as to whether uric acid binds to albumin. There are some works where this fact has been confirmed [56,57,58,59]. It has been shown that the binding rate of pure uric acid is 4% with 5% BSA and approximately 7% in the case of normal heparinized plasma under the same conditions [56].

To sum up, UV irradiation does not affect the antioxidant properties of uric acid, and uric acid does not serve as a UV filter protecting albumin from UV damage. It is most likely that this effect is due to the peculiarities of UV absorption by aqueous solutions of the major components of biological fluids: albumin and uric acid. Figure S2 (see Supplementary Materials) shows the absorption spectra of individual solutions of uric acid and albumin at pH 7.4. Although uric acid (Figure S2a) is absorbed at 10 times the concentration of protein, the albumin solution shows large absorbance values (Figure S2b). The vast majority of bioactive compounds are bound to serum albumin and are transported by it in the bloodstream in a bound state. Among these bioactive substances, uric acid is one of the important ligands. Using bovine serum albumin as an example, it has been demonstrated that the interaction of albumin with uric acid causes a variety of changes in the tertiary structure of the protein [58]. These changes affected tryptophan and tyrosine residues; in particular around Trp residues, there was folding of polypeptide chains [58]. The authors hypothesized that excessive amounts of uric acid can cause disruption of the physiological functions of albumin.

#### 2.1.7. Antioxidant Profile of Blood Plasma after UV Irradiation

**Figure 9:**
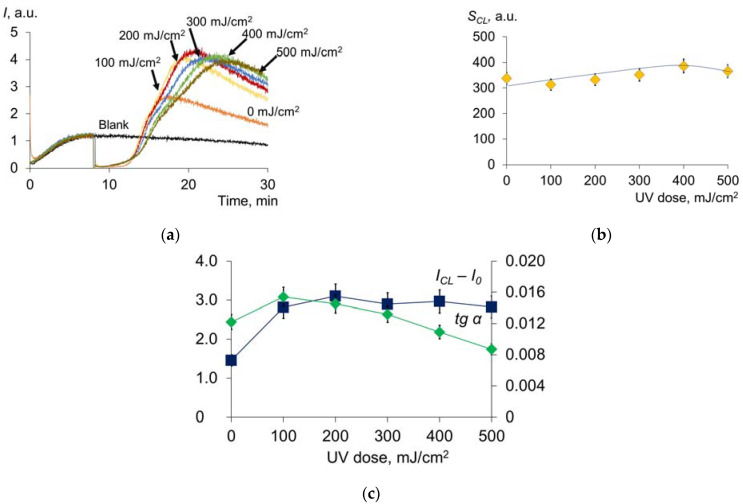
(**a**) Antioxidant profile of blood plasma of a healthy donor after exposure to UV radiation (254 nm; doses are shown in the figure) under the system of PBS (100 mM, pH 7.4) + ABAP (2.5 μM) + luminol (2.0 μM); (**b**,**c**) dependence on *S_CL_*, *I_CL_* − *I*_0_, *tg α* of UV doses (shown in the figure).

For donor blood plasma, based on which the model mixture was developed in the previous section, UV irradiation was performed at different doses (Figure 9a):

Compared with the model aqueous solution of albumin and uric acid, the rh composition of plasma is more complex, which accounts for the difference in the antioxidant profiles of plasma and the model mixture. In contrast to the UA + HSA mixture, irradiation of the plasma showed virtually no change in the chemiluminescence suppression area (Figure 9b), reflecting the contribution to the antioxidant capacity of the strong antioxidants. The *I_CL_* − *I*_0_ parameter increases sharply and decreases smoothly (Figure 9c), as does the *tg α* parameter (Figure 9c, comparable with Figure 3). Thus, oxidative damage is weaker compared to pure albumin or a mixture of albumin and uric acid. Therefore, there are components in the plasma (in addition to uric acid) that protect albumin from UV. These are probably lipids or globulins.

In general, the antioxidant profile makes it possible to assess changes in the antioxidant potential of blood plasma as a result of UV irradiation. This can be a basis for selecting an individual dose for a patient in terms of safety.

### 2.2. Pulsed Xenon Lamp (Broadband Spectrum)

**Figure 10:**
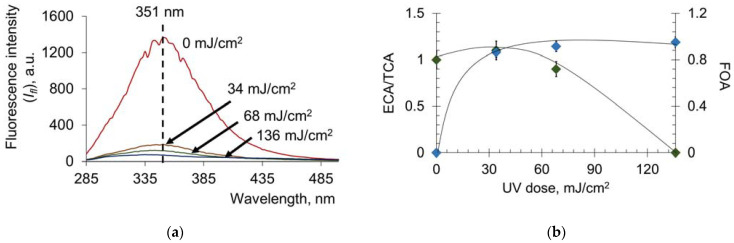
(**a**) Fluorescence spectra of HSA (*c* = 6.6 μM) after exposure to UV (254 nm; doses are shown in the figure); (**b**) increase in ECA/TCA and FOA depending on the UV dose. Registration of fluorescence at λ_ex_ = 260 nm.

Next, we investigated the effect of a new promising UV source, the flash xenon lamp, on the oxidative modification, transport and antioxidant properties of HSA. According to spectrofluorimetric analysis, 50% oxidative modification was achieved at lower doses than for the mercury lamp (approximately 10 mJ/cm^2^ compared with 300 mJ/cm^2^) (Figure 10a,b). At the same time, the reduction in ECA/TCA starts at a dose of 30 mJ/cm^2^, whereas for the mercury lamp, this value is 300 mJ/cm^2^. Therefore, the comparable effects of conformational changes are achieved for doses that are an order of magnitude lower in the case of the flash xenon lamp compared to the mercury lamp.

**Figure 11:**
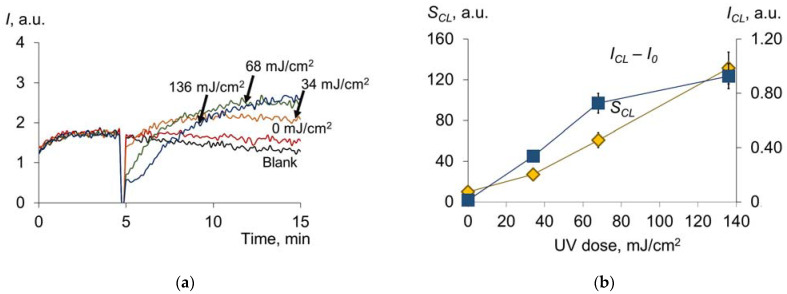
(**a**) Chemiluminograms of HSA (160 nM) after exposure to UV (254 nm; doses are shown in the figure) under the system of PBS (100 mM, pH 7.4) + ABAP (2.5 μM) + luminol (2.0 μM); (**b**) dependence of *I_CL_* − *I*_0_ and the *S_CL_* on the UV dose.

When studying the antioxidant profile of UV-modified HSA (Figure 11a), we can see changes in *I_CL_* − *I*_0_ and *S_CL_* (Figure 11b), such as those obtained for the mercury lamp (see Figure 3c,d):

For pulsed broadband irradiation, the qualitative trend of increasing antioxidant properties is demonstrated, such as for the mercury lamp. However, there are quantitative differences. At a dose of 136 mJ/cm^2^ for the Xe lamp, *I_CL_* − *I*_0_ = 0.93 arb.u., *S* = 131 arb.u.; for the Hg lamp, the same values will be achieved at doses equal to 625 mJ/cm^2^ and 35 mJ/cm^2^, respectively. According to the data obtained, approximately four times higher UV doses from a mercury lamp compared to a xenon lamp are required to achieve comparable antioxidant capacity. It is interesting to note that the 250–260 nm range accounts for 0.97 of the total radiation energy of the xenon source, and the total bactericidal range accounts for 8.35% of the total radiation. Thus, we can assume that most of the effects of UV radiation on albumin are not caused by the bactericidal band but by the rest of the band.

The studies mainly compare the effects of pulsed and continuous UV radiation on various micro-organisms for the purpose of disinfection. However, there are no systematic studies of these sources on individual biomolecules in the body. For example, in Ref. [60], it was shown that pulsed UV radiation inactivates bacteria, fungi, viruses faster and more effectively compared to continuous radiation. The antimicrobial effects are explained by chemical modification and breaks of DNA chains in the target cells, including photodimerization, photohydration, cross-linking with proteins, etc. Since UV treatment is used to inactivate micro-organisms, in particular the most infectious pathogens of food origin, a question arose regarding possible conformational changes of food components when exposed to pulsed radiation. In Ref [61], the authors investigated the protein composition of milk using UV spectroscopy, spectrofluorimetry, electrophoresis and determination of the amino acid composition. According to the results obtained, when β-lactoglobulin was exposed to pulsed UV radiation, there was a slight change in the polarity of the microenvironment of the tryptophanyl residue and, most likely, aggregation of the protein. No structural abnormalities in the amino acid composition or oxidation products were observed. The authors concluded that there were no significant conformational changes in milk proteins despite aggregation due to disulfide bonds [61]. A later study [62] investigated a similar problem using whey protein isolate as an example. The main components of serum protein are β-lactoglobulin, α-lactoglobulin, bovine serum albumin and immunoglobulins. The treatment of the serum protein isolate with pulsed UV radiation caused structural changes, as evidenced by a gradual increase in the content of free thiol groups (unfolding of the molecule) and the subsequent formation of a small fraction of aggregated proteins due to hydrophobic interactions and disulfide bonds [62].

An important method of the effect of UV irradiation on the conformation, stability and binding properties of protein molecules is circular dichroism spectroscopy (CD spectroscopy) [9,63]. Using BSA as an example, according to CD spectra, it was shown that UV irradiation leads to a decrease in the ordering of the protein structure [64]. In particular, the information obtained mainly indicates the secondary structure of the protein; namely, UV irradiation can destabilize the helical structure [65]. Studying the albumin structure using CD spectroscopy should be the next step of our work.

The results of our study indicate that exposure to both continuous linear UV radiation and pulsed broadband leads to qualitatively similar effects on human serum albumin, but quantitatively, the xenon lamp is a more effective source. An order of magnitude lower doses are required to achieve the same effect. Further study of the effect of pulsed UV radiation on biomolecules and blood plasma for selecting therapeutically effective wavelength range and dose is promising for theoretical and practical photomedicine. The proposed methodological approach allows us to compare oxidative modification of albumin, one of the main proteins of blood plasma, after exposure to different sources of UV radiation and to find those doses, which are harmless.

## 3. Materials and Methods

### 3.1. Reagents and Samples

Stock solutions of human serum albumin (HSA, Sigma-Aldrich, St. Louis, MO, USA), L-tyrosine (Tyr, Acros Organics, Morris Plains, NJ, USA), L-tryptophan (Trp, Sigma-Aldrich, USA) and glycyl-L-tryptophan (Gly-Trp, Sigma-Aldrich, USA) were prepared by dissolving weighed portions in a 100 mM phosphate-buffered solution with pH 7.4 (PBS, KH_2_PO_4_, Sigma-Aldrich, USA). A weighed portion of γ-globulins (Sigma-Aldrich, USA) was dissolved in 0.9% NaCl (solution for infusion, Asfarma Ltd., Moscow, Russia). A stock solution of uric acid (UA, Fluka, New York, NY, USA) was prepared in 32 mM KOH (Sigma-Aldrich, USA); the working solution was prepared by diluting the initial PBS with subsequent pH adjustment to 7.4.

Blood plasma was obtained from a healthy donor in a clinical laboratory on the day of the experiment. Blood (≈6 mL) was drawn into Li-heparin vacutainers by qualified personnel. The samples were centrifuged in test tubes at 1000× *g* for 10 min. During the day, the plasma was stored at 4 °C. The donor signed informed consent. The design was approved by the Ethics Committee of the Research Centre for Medical Genetics (Protocol #5, May 2019).

### 3.2. Ultraviolet Sources

#### 3.2.1. Mercury Lamp UV

Working solutions (absorbance ≤ 0.2) of HSA, Tyr, Trp, Gly-Trp, uric acid, γ-globulins and healthy donor plasma were irradiated in a quartz cuvette (volume 3.000 mL) using a Bio-Link UV irradiation system (Vilber Lourmat, Collégien, France) at 254 nm (UV source 5 × 8-watt lamps). The emission spectrum of a mercury lamp UV is presented on the manufacturer’s website: https://www.vilber.com/pdf/leaflet/leaflet-uv-instruments.pdf (accessed on 1 September 2016). The radiation dose for a mercury lamp UV was calculated using TracePro 7.1.5 Expert software.

#### 3.2.2. Flash Xenon UV

**Figure 12:**
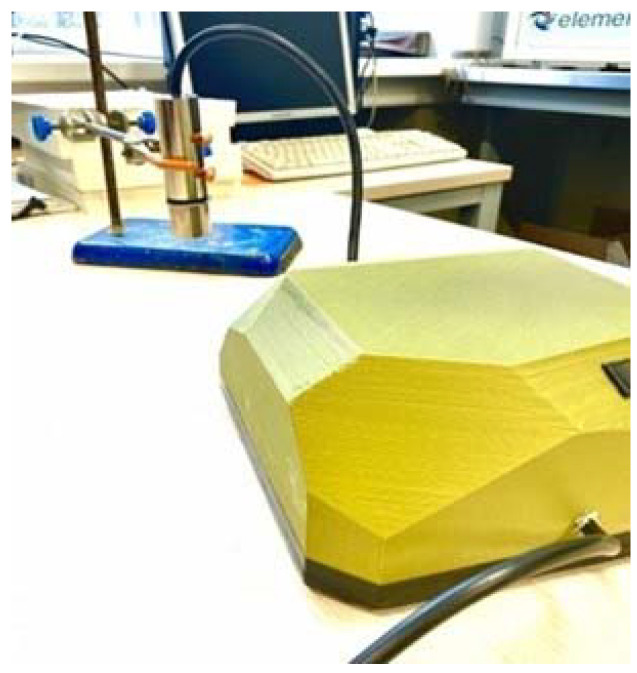
The therapeutic device model.

**Figure 13:**
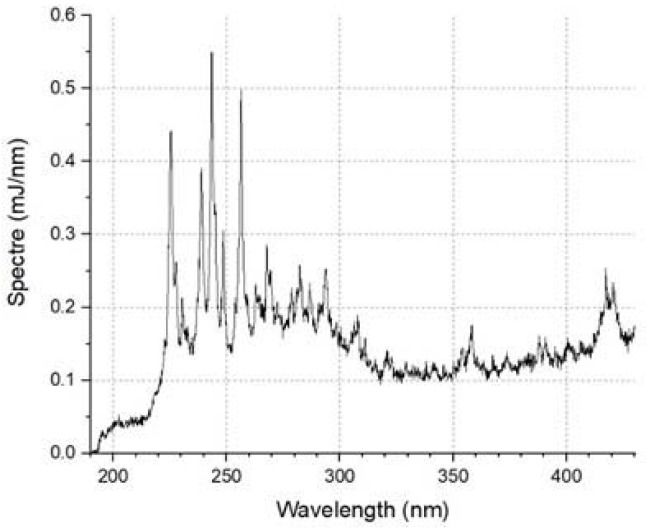
The emission spectrum of the Hamamatsu L11937 lamp.

**Figure 14:**
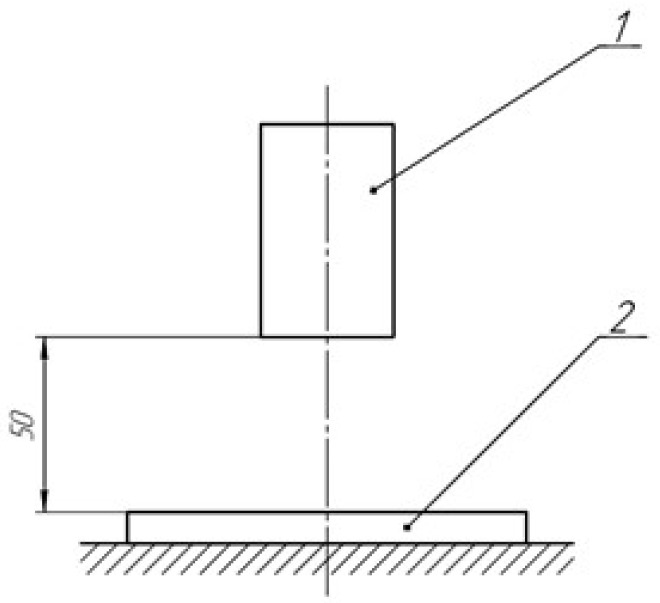
Schematic of the experiment: 1—UV lamp; 2—Petri dish with a sample.

As a source of high-intensity flash radiation of a wide spectrum, we used the therapeutic device model (Figure 12) based on a flash ball lamp L11937 (Hamamatsu, Japan):

The power of the device was 30 W, and the nominal lamp power was 20 W; the average radiation power in the range of 200–300 nm was 0.357 W. The flash ball lamp emission spectrum is shown in Figure 13:

The HSA solution (5.000 mL) was irradiated in a Petri dish, as shown in Figure 14. The thickness of the liquid layer in the Petri dish was no more than 2 mm, which provided uniform irradiation in depth.

The distance from the UV source to the samples during irradiation was 5 cm. The radiation dose received by the sample in 1 s was calculated as the radiation dose from a point source, and it was 0.113 mJ/cm^2^∙s.

### 3.3. Oxidative Modification and Transport Capacity of Albumin

Analysis of the oxidative modification of albumin and its transport properties was performed with spectrofluorimetry. Fluorescence spectra were recorded in quartz cuvettes (*l* = 1.00 cm, *V* = 3.000 mL) on an RF-5301 PC spectrofluorimeter (SHIMADZU, Kyoto, Japan). The width of the entrance and exit slits of the monochromator is 3 nm; the scanning step is 0.2 nm. The signal-to-noise ratio is no less than 150 for the Raman line of distilled water (λ_ex_ = 350 nm; the slit width is 10 nm).

#### 3.3.1. Assessment of Oxidized Albumin Fraction

To quantify oxidized albumin, we used a previously developed protocol [39]. The protocol consists of recording the fluorescence spectra of UV-modified HSA at 260 nm (I) and calculating the fraction of oxidized albumin (FOA) using the following formula:

FOA = (*I*_0_ − *I*)/*I*_0_ (1)

where *I*_0_—calculated (theoretical) fluorescence determined from the calibration graph; *I*—the experimental intensity.

#### 3.3.2. Albumin Transport Capacity

The assay is based on its specific interaction of albumin with the fluorescent probe K-35 (N-(carboxyphenyl)imide of 4-(dimethylamino)naphthalic acid, synthesized in the Federal Research and Clinical Center of Physicochemical Medicine, Ministry of Health of the Russian Federation) [66,67,68,69,70]. The reaction with K-35 allows for selective study of changes in drug site II in the albumin molecule next to which the tryptophan residue is localized. According to this protocol, the effective concentration of albumin (ECA) is calculated as the ratio of *I_fl_* HSA before irradiation to *I_fl_* HSA after irradiation in the presence of a K-35 probe at 518 nm (Figure S3, Supplementary Materials); the registration of fluorescence spectra is carried out at 445 nm. The ECA/TCA ratio is used as an analytical parameter, where TCA is a total concentration of albumin.

### 3.4. Assessment of Thiol Groups

The determination of thiol groups (-SH) in albumin after exposure to UV was performed using a standard commercial Fluorometric Thiol Assay Kit (Sigma-Aldrich, USA). Freshly prepared protein solutions were used for no more than 2 h.

### 3.5. Antioxidant Capacity Assay

A model reaction of alkylperoxyl radical generation in the decomposition of 2,2′-azobis(2-amidinopropane) dihydrochloride (ABAP) was used to analyze the antioxidant properties. Briefly, ABAP (2.5 μM, #123072, Sigma, St. Louis, MO, USA) and luminol (2.0 μM, #123072, Sigma) were added to a cuvette with PBS (100 mM, pH 7.4) thermostated at 37 °C. The method was described elsewhere [71]. After the chemiluminescence intensity reached a stationary value, an aliquot of the studied solution was added.

Chemiluminescence was recorded on a 12-channel Lum-1200 chemiluminometer (DISoft, Russia) for at least 20 min. The data were processed using PowerGraph software (version 3.3).

## 4. Conclusions

To date, the search for highly effective methods of treatment of socially significant diseases continues. To this end, ultraviolet blood irradiation (UVBI) is of particular interest due to the described therapeutic effects, including as part of combination therapy with various drugs. In addition, UVBI can be considered as a new immunomodulatory method. Despite the promising potential of UV therapy, there are no data on the molecular mechanisms of the interaction of therapeutic doses of UV radiation with blood components.

In this work, we studied the oxidative effects of exposure to low doses of UV radiation on the major water-soluble components of the blood: albumin, globulins and uric acid. UV irradiation leads to conformational changes in albumin accompanied by an attenuation of transport properties. At the same time, UV irradiation leads to marked enhancement of the antioxidant properties of albumin and globulins. It was found that uric acid has no protective properties with respect to UV irradiation of albumin. The effect of a promising source of UV radiation for UVBI (xenon lamp) on the major protein of blood plasma was demonstrated. Pulsed broadband irradiation (xenon lamp) has qualitatively the same effects on albumin as continuous linear irradiation (mercury lamp), but an order of magnitude lower doses are required to achieve comparable effects. Since the bactericidal range accounts for 8.35% of the total radiation energy of the xenon source, it can be assumed that the main contribution to the effect of UV radiation on albumin is not bactericidal range. Registration of blood plasma antioxidant profile through luminol-dependent chemiluminescence with ABAP can be used to assess the effect of UV irradiation on plasma albumin and the basis for selection of individual therapeutic dose. In the future, more detailed studies of the effect of pulsed broadband radiation are required, since the dose dependence is different from the conventional mercury source.

The results achieved in this work are important from a biomedical point of view. They may contribute to the understanding of the fundamental mechanisms of the action of UV radiation on the blood and, accordingly, the feasibility of using UV radiation for the treatment of various diseases. In addition, the data obtained allow us to assess the possible biological consequences of UV irradiation of the blood.

## Figures and Tables

**Table 1:** Content of free SH groups in human serum albumin after exposure to UV (254 nm).

UV Dose, mJ/cm^2^	Thiols (μM)
100	1.63 ± 0.01
200	3.95 ± 0.03
300	5.19 ± 0.04
400	6.48 ± 0.09
500	7.89 ± 0.08

## Data Availability

All inquiries can be directed to the corresponding authors.

## Supplementary Materials

The following supporting information can be downloaded at: https://www.mdpi.com/article/10.3390/molecules28124646/s1, Figure S1: (a) Chemiluminograms of the Trolox under the system of PBS (100 mM, pH 7.4) + ABAP (2.5 μM) + luminol (2.0 μM); (b) dependence of parameter *S_CL_* (a.u.) on the Trolox concentrations (nmol/L); Figure S2: Absorption spectra of (a) uric acid (30 μM) and (b) albumin (3 μM) in PBS (100 mM, pH 7.4); *l* = 1 cm; Figure S3: Fluorescence spectra of albumin (6.6 μM) after UV irradiation, recorded in the presence of a K-35 fluorescent probe (λ_ex_ = 445 nm) and the fluorescence spectrum of an individual solution of K-35.
