# Supplementary material for: Effect of Low-Dose Line-Spectrum and Full-Spectrum UV on Major Humoral Components of Human Blood
Source: Molecules. 2023 Jun 8;28(12):4646. doi: 10.3390/molecules28124646 (PMC10304147; doi:10.3390/molecules28124646)
Supplement: Supplementary file 1 [file molecules-28-04646-s001.zip › molecules-2363330-supplementary.pdf]

molecules

MDPI

Supplementary Materials

of

Effect of Low-Dose Line-Spectrum and Full-Spectrum UV on Major Humoral Components of Human Blood

by

Madina M. Sozarukova [1,*], Nadezhda A. Skachko [2], Polina A. Chilikina [2], Dmitriy O. Novikov [2] and Elena V. Proskurnina [3]

1 Kurnakov Institute of General and Inorganic Chemistry, Russian Academy of Sciences, Leninsky av., 31, 119991 Moscow, Russia
2 Department of Plasma Power Plants, Bauman Moscow State Technical University, 2-nd Baumanskaya, 5, 105005 Moscow, Russia; nadyans96@mail.ru (N.A.S.); p.chilikina@gmail.com (P.A.C.); zerooo@list.ru (D.O.N.)
3 Research Centre for Medical Genetics, ul. Moskvorechye 1, 115522 Moscow, Russia; proskurnina@gmail.com
* Correspondence: s_madinam@bk.ru; Tel.: +7-(926)-724-8286

Quantification of the antioxidant properties of albumin

The antioxidant activity of the albumin solution with a concentration of 1 μmol/l was evaluated in terms of the concentration of the water-soluble analog of vitamin E, Trolox. Previously, for an aqueous solution of Trolox, chemiluminograms were recorded under the system with alkylperoxyl radicals (Figure S1a). Using data from the chemiluminescent analysis, the experimental dependence of the parameter *S* (antioxidant capacity, arb.u.) on the concentration of the Trolox solution was determined (Figure S1b).

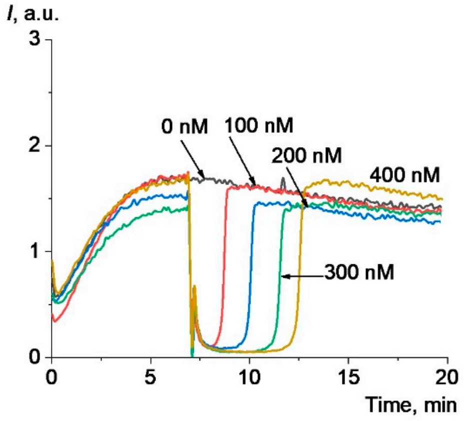

(**a**)

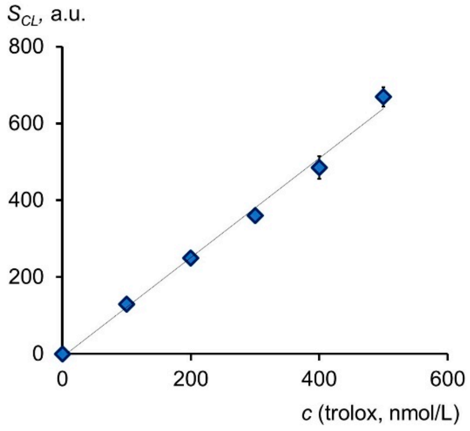

(**b**)

Figure S1. (**a**) Chemiluminograms of the Trolox under the system of PBS (100 mM, pH 7.4) + ABAP (2.5 μM) + luminol (2.0 μM); (**b**) dependence of parameter S_{CL} (a.u.) on the Trolox concentrations (nmol/L).

The equation of the calibration line: $S = (1.30 \pm 0.04) \times c$ (Trolox, nmol/L) – (7.40 ± 0.04), $r = 0.998$, $P = 0.95$, $n = 6$, S_{CL} = area of chemiluminescence suppression. Using the values of the S_{CL} parameter,

the antioxidant capacity of a 1 μmol/L albumin solution after exposure to a dose of 100 mJ/cm^2 was estimated in terms of the concentration of the Trolox, μmol/L. According to the estimates, the ability of albumin irradiated with a dose of 100 mJ/cm^2 to scavenge free radicals is, on average, three times lower than that of Trolox.

Absorption spectra of uric acid and albumin

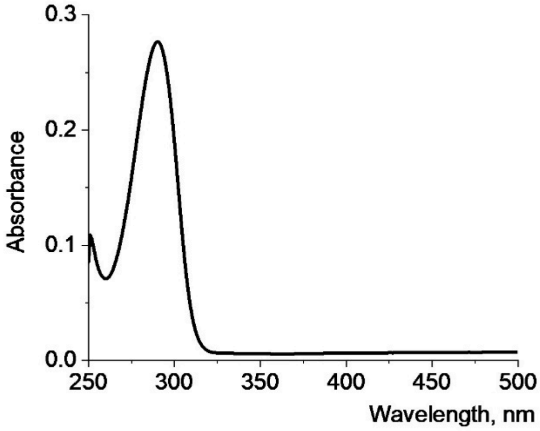

(**a**)

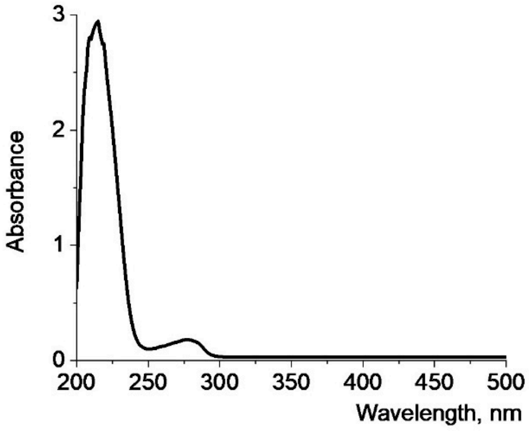

(**b**)

Figure S2. Absorption spectra of (**a**) uric acid (30 μM) and (**b**) albumin (3 μM) in PBS (100 mM, pH 7.4); l = 1 cm.

Fluorescence spectra of albumin in the presence of probe K-35

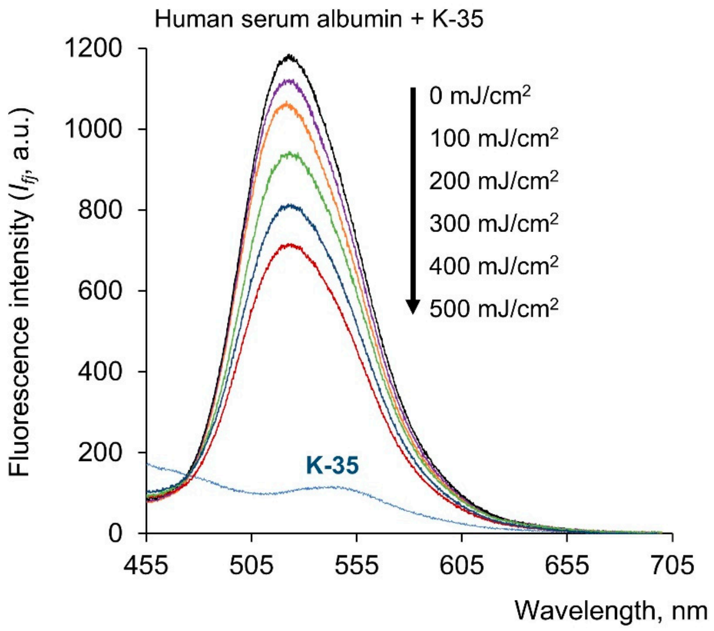

Figure S3. Fluorescence spectra of albumin (6.6 μM) after UV irradiation, recorded in the presence of a K-35 fluorescent probe (λ_{ex} = 445 nm) and the fluorescence spectrum of an individual solution of K-35.
